# Supplementary figures and images for: Maintenance of proteostasis by Drosophila Rer1 is essential for competitive cell survival and Myc-driven overgrowth
Source: PLoS Genet. 2024 Feb 26;20(2):e1011171. doi: 10.1371/journal.pgen.1011171 (PMC10919865; doi:10.1371/journal.pgen.1011171)

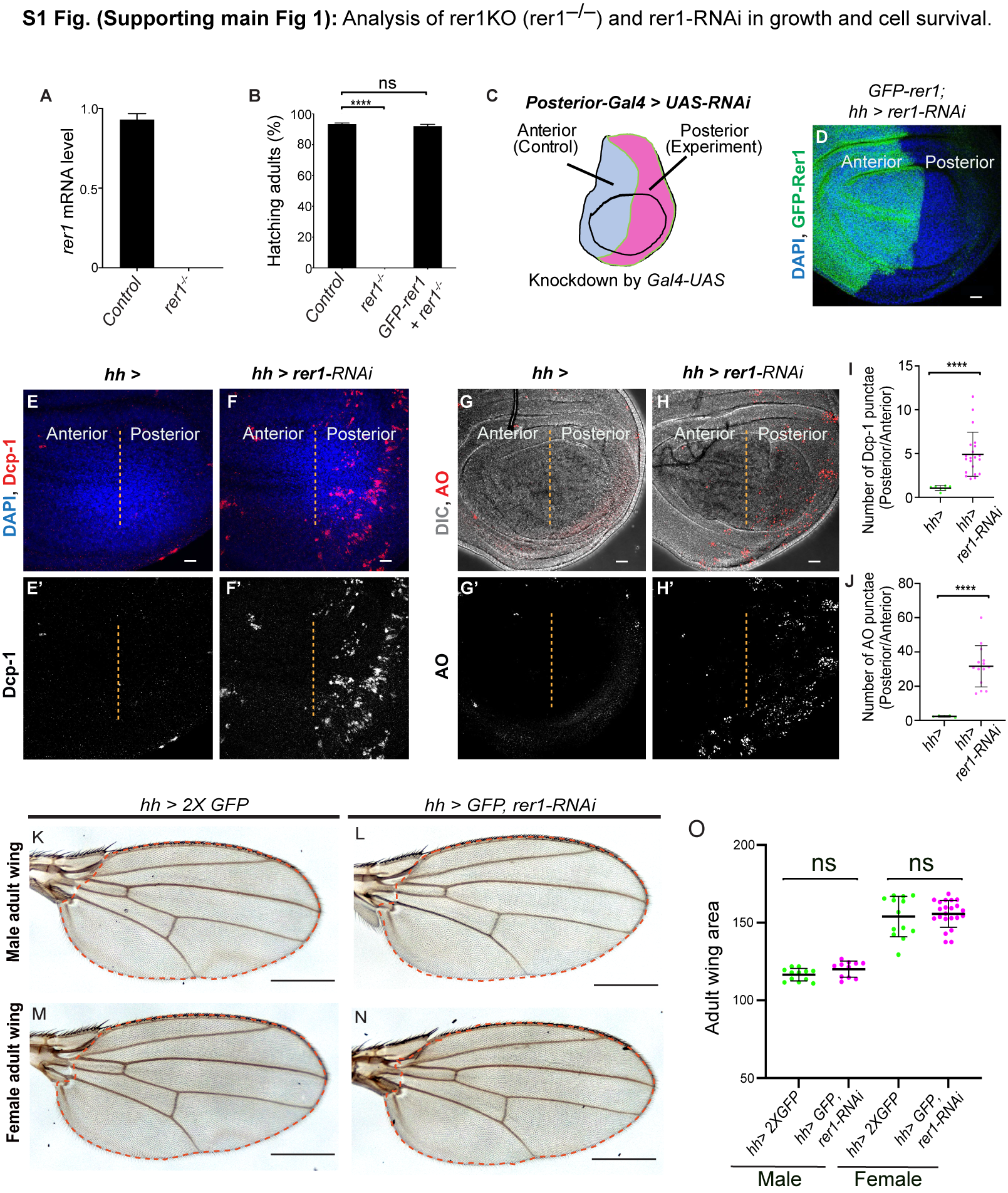

Supplement: S1 Fig — (A) rer1 mRNA expression levels were measured by quantitative PCR in rer1KG08816 (control, precise excision) and homozygous rer1KO (rer1–/–) flies. Bars show mean±SEM (N = 3 independent experiments). (B) rer1–/–flies failed to hatch out. Most of them died before the pupae stage. Re-introducing the rer1 genomic fragment (GFP-rer1) rescued the phenotype, underscoring that they are caused by rer1 deficiency. Statistical analyses in B were performed using the Ordinary one-way ANOVA with Dunnett’s multiple comparison test, (**** p<0.0001). (C) Scheme of a wing disc illustrating anterior and posterior compartments in which transgene was expressed with a posterior specific Gal4. (D) hh-Gal4 mediated depletion of Rer1 in GFP-rer1 background [GFP-rer1; hh-Gal4::UAS-rer1-RNAi] shows loss of GFP signal in the posterior compartment. (E–F) Dcp-1 staining on (E-E’) control (hh-Gal4, N = 7 wing discs) and (F-F’) Rer1 depleted (hh-Gal4, rer1-RNAi, N = 21 wing discs) wing discs. (G–H) Acridine Orange (AO) staining on (G-G’) control (hh-Gal4, N = 5 wing discs) and (H-H’) Rer1 depleted (hh-Gal4, rer1-RNAi, N = 14 wing discs) wing discs. (I-J) Quantification of Dcp-1 punctae (I) and AO punctae (J) numbers in the posterior compartments of either control or Rer1 depleted discs, normalized to their respective anterior compartments. Statistical analysis was performed using two-tailed Welch’s t-test (**** p<0.0001). SB = 20 μm. (K-N) Adult wings from control flies (hh-Gal4::2x UAS-GFP); male, K, N = 12 and female, M, N = 13) and flies harboring hh-Gal4 mediated Rer1 knockdown along with overexpression of GFP (hh-Gal4::UAS-rer1-RNAi, UAS-GFP); male, L, N = 11 and female, N, N = 22). (O) Quantification of the adult wing areas measured within the dotted line. Statistical analysis was performed using two-tailed Welch’s t-test. P values for male wing comparison (K-L) was p = 0.0793, for female wing comparison (M-N) was p = 0.6704. SB = 500 μm. (TIF) [file pgen.1011171.s001.tif]

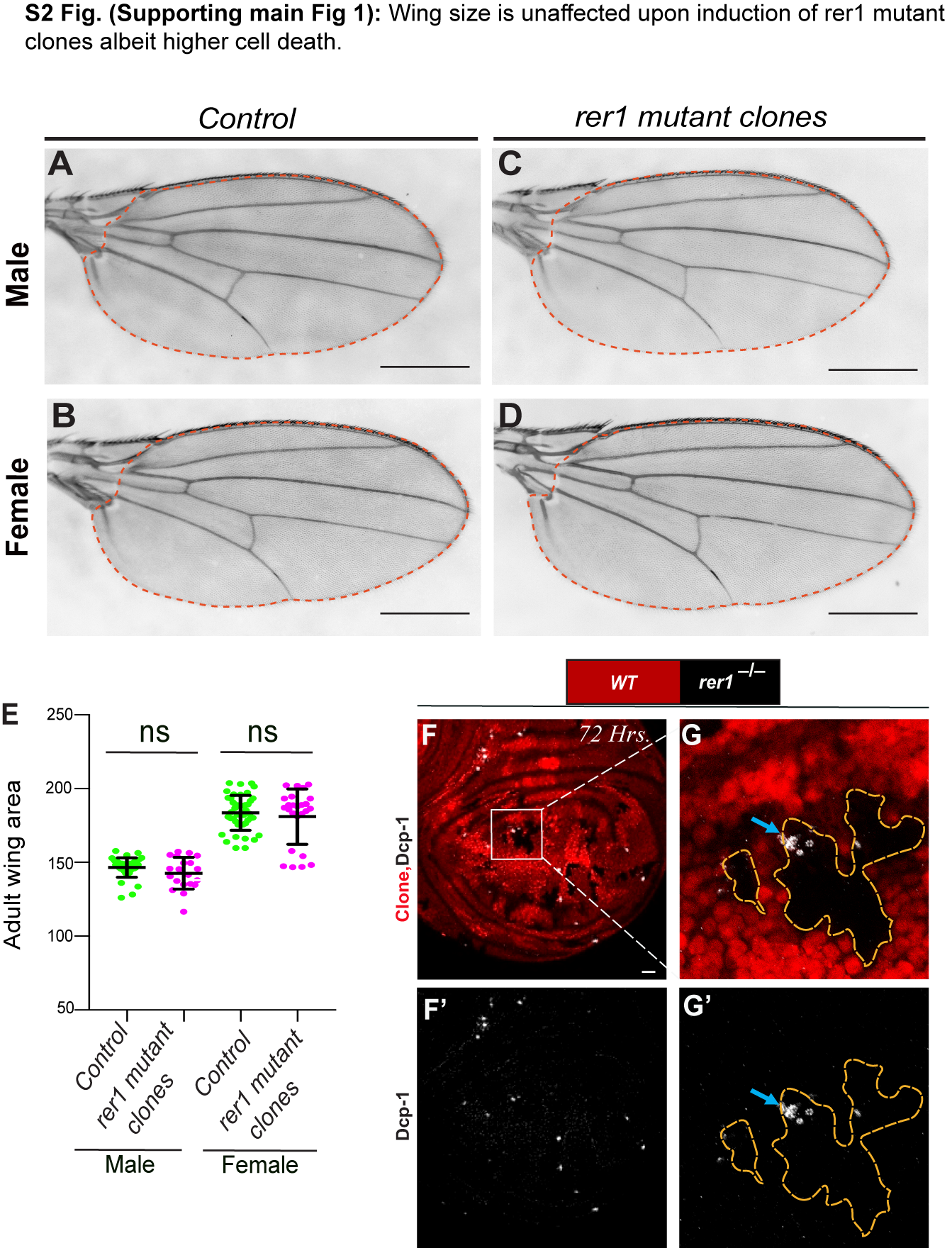

Supplement: S2 Fig — (A-B) Images of adult wings from control flies harboring wild-type clones induced at 48hrs AEL (A, male of genotype hs-FLP/Y;; FRT82B, Ubi-RFP.nls/ FRT82B, Ubi-GFP.nls; N = 39 and B, female of genotype hs-FLP/+;; FRT82B, Ubi-RFP.nls/ neoFRT 82B, ry506, rer1KO; N = 44). (C-D) Images of adult wings from flies with rer1 mutant clones induced at 48hrs AEL (C, males of genotype hs-FLP/ Y;; FRT82B, Ubi-RFP.nls/ FRT82B, Ubi-GFP.nls; N = 20 and D, females of genotype hs-FLP/+;; FRT82B, Ubi-RFP.nls/ neoFRT82B, ry506, rer1KO; N = 25). (E) Quantification of the adult wing area measured within the dotted line (Two-tailed Welch’s t-test). P values for male wings size comparison (A-C) was P = 0.1494, for female wings size comparison (B-D) was P = 0.5407. SB = 500 μm. (F-G) Wing imaginal disc harboring RFP negative rer1–/–clones (72 hrs AHS), stained with anti Dcp-1 antibody to show the cell death at clone boundary. (G) A magnified image of the white inset in F. SB = 20 μm. (TIF) [file pgen.1011171.s002.tif]

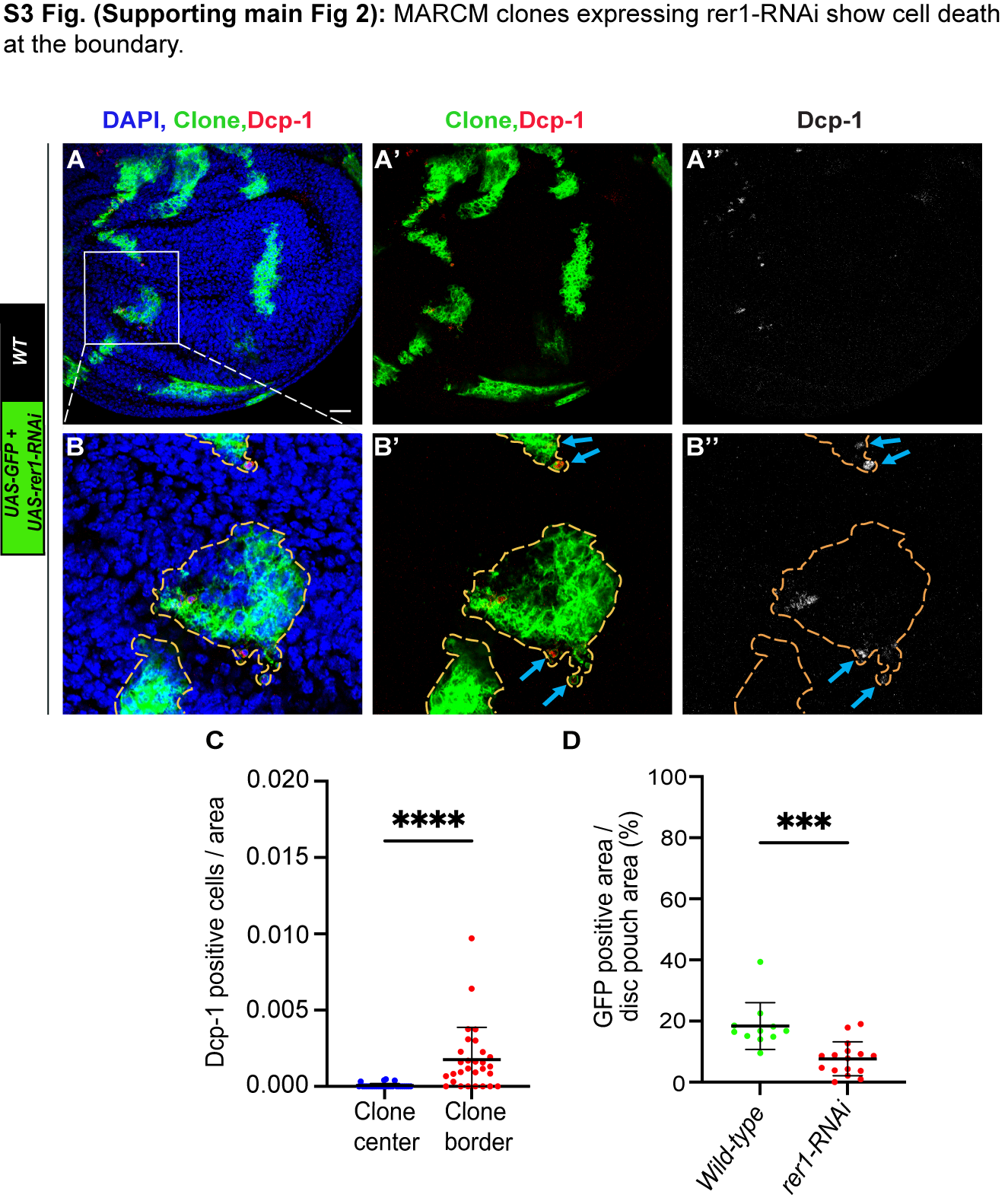

Supplement: S3 Fig — (A-A”) Representative images of the wing discs containing hs-FLP induced rer1-RNAi expressing MARCM clones (72 hrs AHS), stained with anti-Dcp-1 antibody. (B-B”) Magnified images of the white box in A. (C) Quantification of Dcp-1 positive cells at the center and border of rer1-RNAi clones (N = 29 clones in 13 wing discs); two-sided Wilcoxon signed-rank test, **** p<0.0001. (D) Quantification of GFP positive clone area in wing disc harboring wild-type (N = 11 wing discs) and rer1-RNAi (N = 16 wing discs) MARCM clones (Two-tailed Welch’s t-test). SB = 20 μm. *** p = 0.0009. (TIF) [file pgen.1011171.s003.tif]

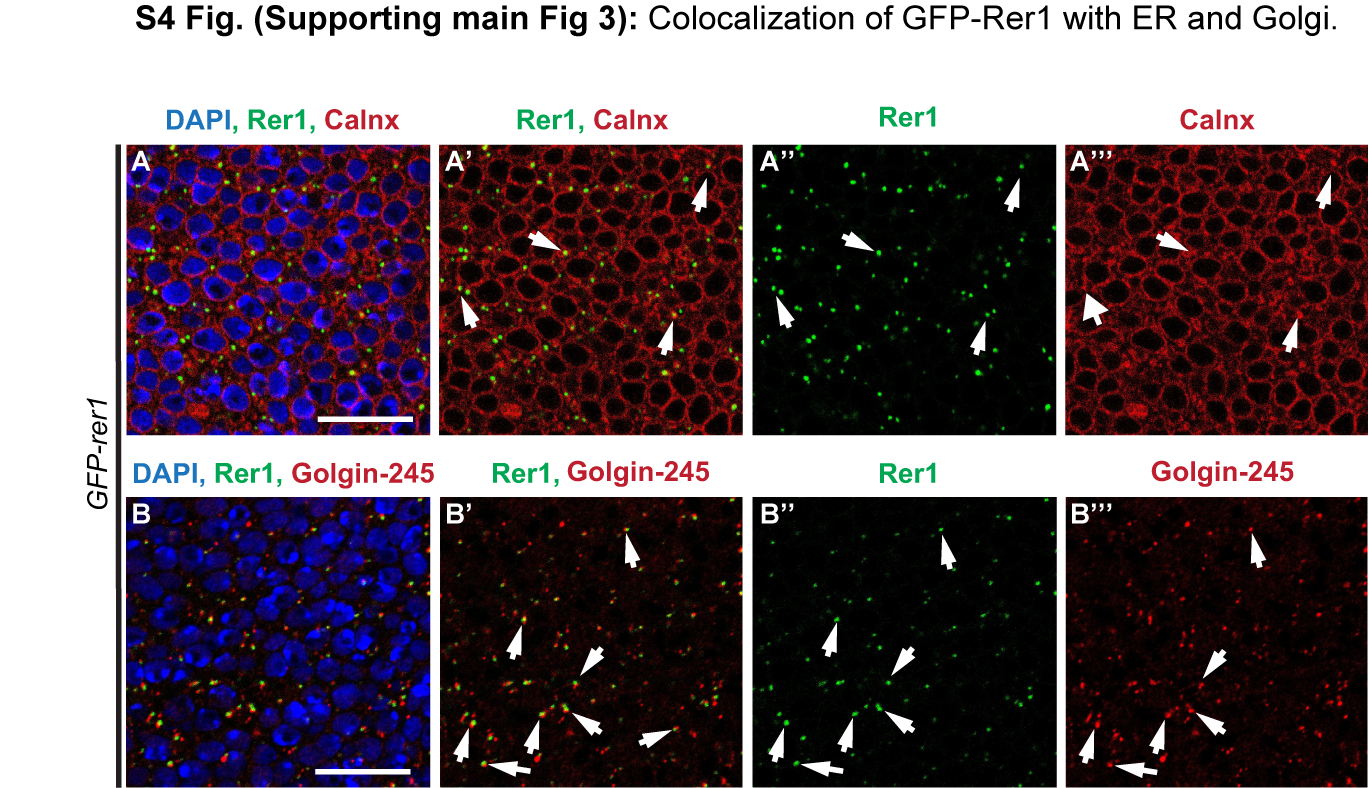

Supplement: S4 Fig — (A-A”’) Colocalization of GFP-tagged Rer1 and ER, stained with Calnexin (red). (B-B”’) Colocalization of GFP-tagged Rer1 and Golgi, marked by the Golgin-245 (red). White arrows showed the colocalized punctae. SB = 20 μm. (TIF) [file pgen.1011171.s004.tif]

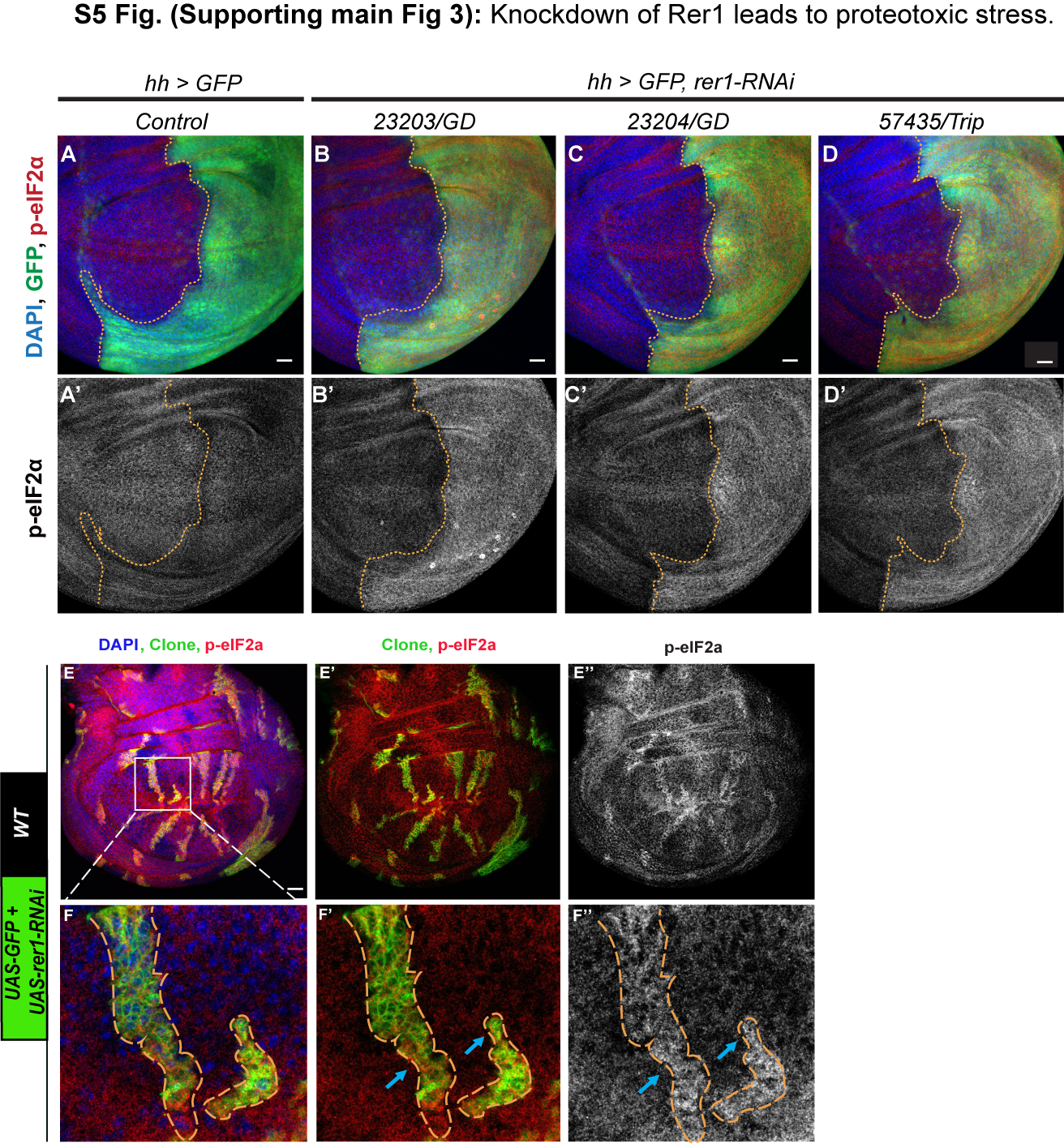

Supplement: S5 Fig — (A) Control wing disc (hh-Gal4::UAS-GFP, N = 5) showing expression of hh-Gal4 in the posterior compartment, marked by GFP. (B–D) hh-Gal4 mediated depletion of Rer1 (hh-Gal4::UAS-GFP, UAS-rer1-RNAi) in the posterior compartment using three different RNAi lines 23203/GD (B; N = 6 wing discs); 23204/GD (C; N = 8 wing discs) and 57435/Trip (D; N = 6 wing discs), shows an increase in the p-eIF2α level compare to the anterior compartment. (E-F) Images of the wing discs containing hs-FLP induced rer1-RNAi (Trip) expressing MARCM clones (72 hrs AHS; N = 3 wing discs), stained with anti-p-eIF2α antibody. (F-F”) Magnified images of the boxed area in E. SB = 20 μm. (TIF) [file pgen.1011171.s005.tif]

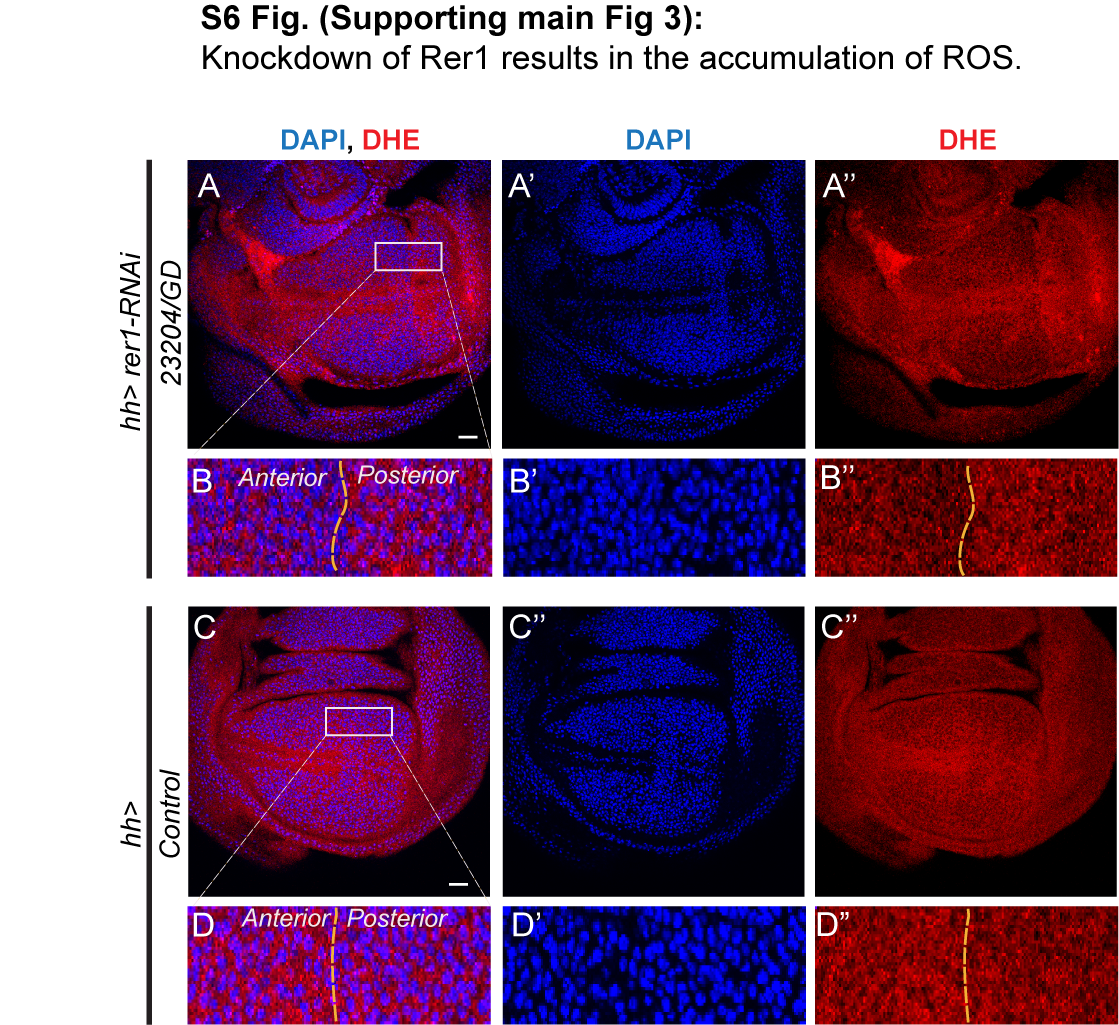

Supplement: S6 Fig — (A-D) DHE uptake assay, indicative of ROS levels, performed on the control and Rer1 depleted wing imaginal discs. (A) Depletion of Rer1 in the posterior compartment [hh-Gal4::UAS-rer1-RNAi] showed higher levels of DHE as compared to the anterior compartment (N = 12 wing discs). (B) Magnified images of the inset in A. (C) control disc [hh-Gal4] showed similar DHE levels between anterior and posterior compartments (N = 8 wing discs). (D) Magnified images of the inset in C. Yellow dotted lines mark the anterior-posterior boundary; Nuclei are stained with DAPI. SB = 20 μm. (TIF) [file pgen.1011171.s006.tif]

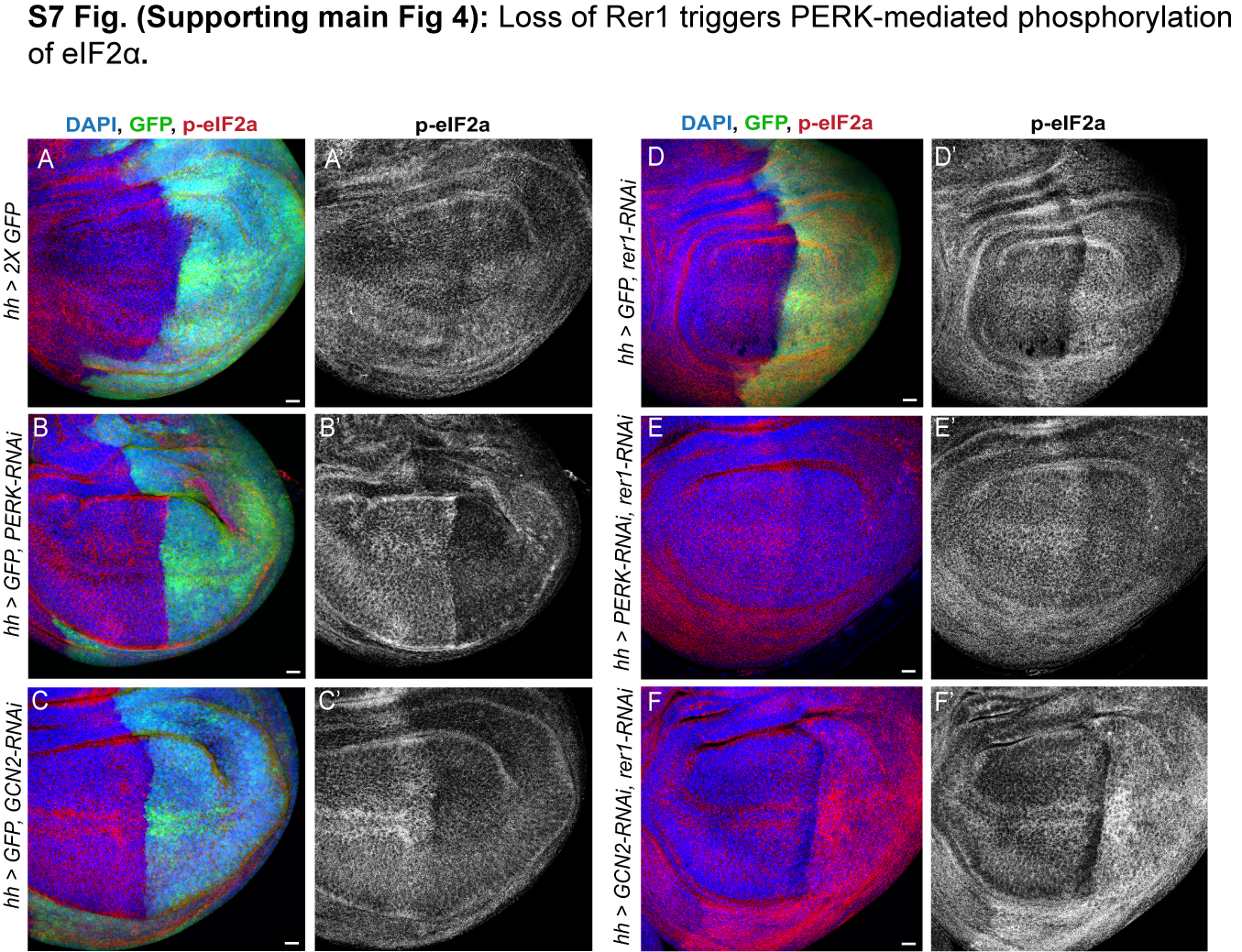

Supplement: S7 Fig — (A-A’) Control third-instar wing disc (hh-Gal4::2X UAS-GFP) stained with anti-p-eIF2α antibody (N = 10 wing discs). (B-D) Third-instar discs with hh-Gal4 mediated coexpression of GFP with PERK-RNAi (B-B’; N = 12 wing discs), GCN2-RNAi (C-C’; N = 5) and rer1-RNAi (D-D’; N = 10 wing discs), stained with anti-p-eIF2α antibody. (E-E’) Disc with coexpression of rer1-RNAi and PERK-RNAi, stained with anti-p-eIF2α antibody (N = 12). (F-F’) Disc with coexpression of rer1-RNAi and GCN2-RNAi, stained with anti-p-eIF2α antibody (N = 13). Nuclei are stained with DAPI. SB = 20 μm. (TIF) [file pgen.1011171.s007.tif]

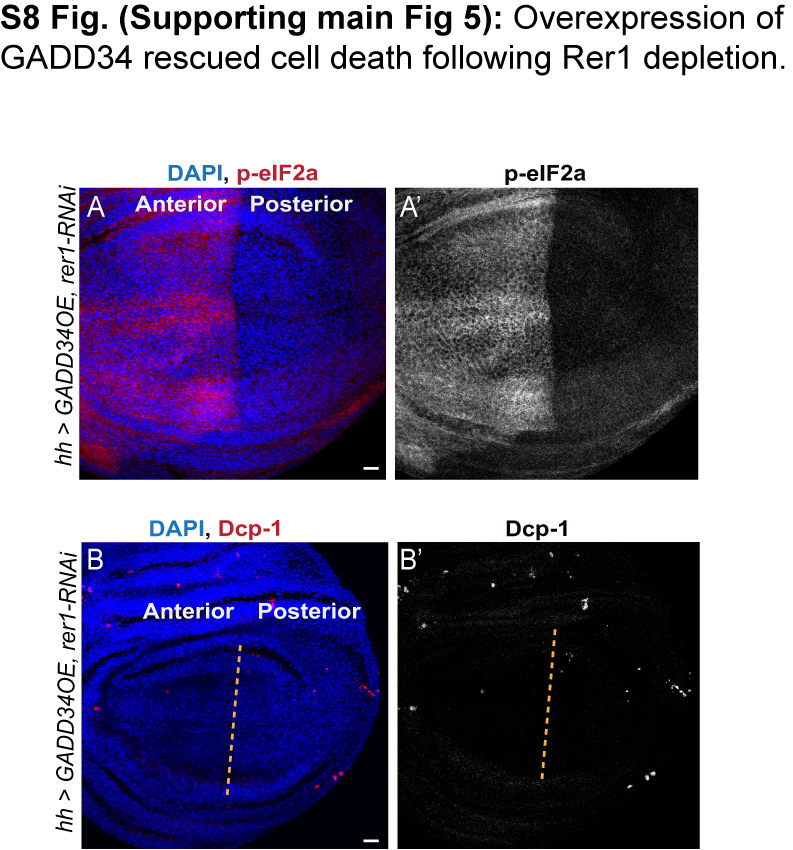

Supplement: S8 Fig — (A-B) Third instar disc with hh-Gal4 mediated overexpression of GADD34 and expression of rer1-RNAi, stained with either anti-p-eIF2α (A; N = 10 wing discs) and anti-cleaved Dcp-1 antibodies (B; N = 15 wing discs). SB = 20 μm. (TIF) [file pgen.1011171.s008.tif]

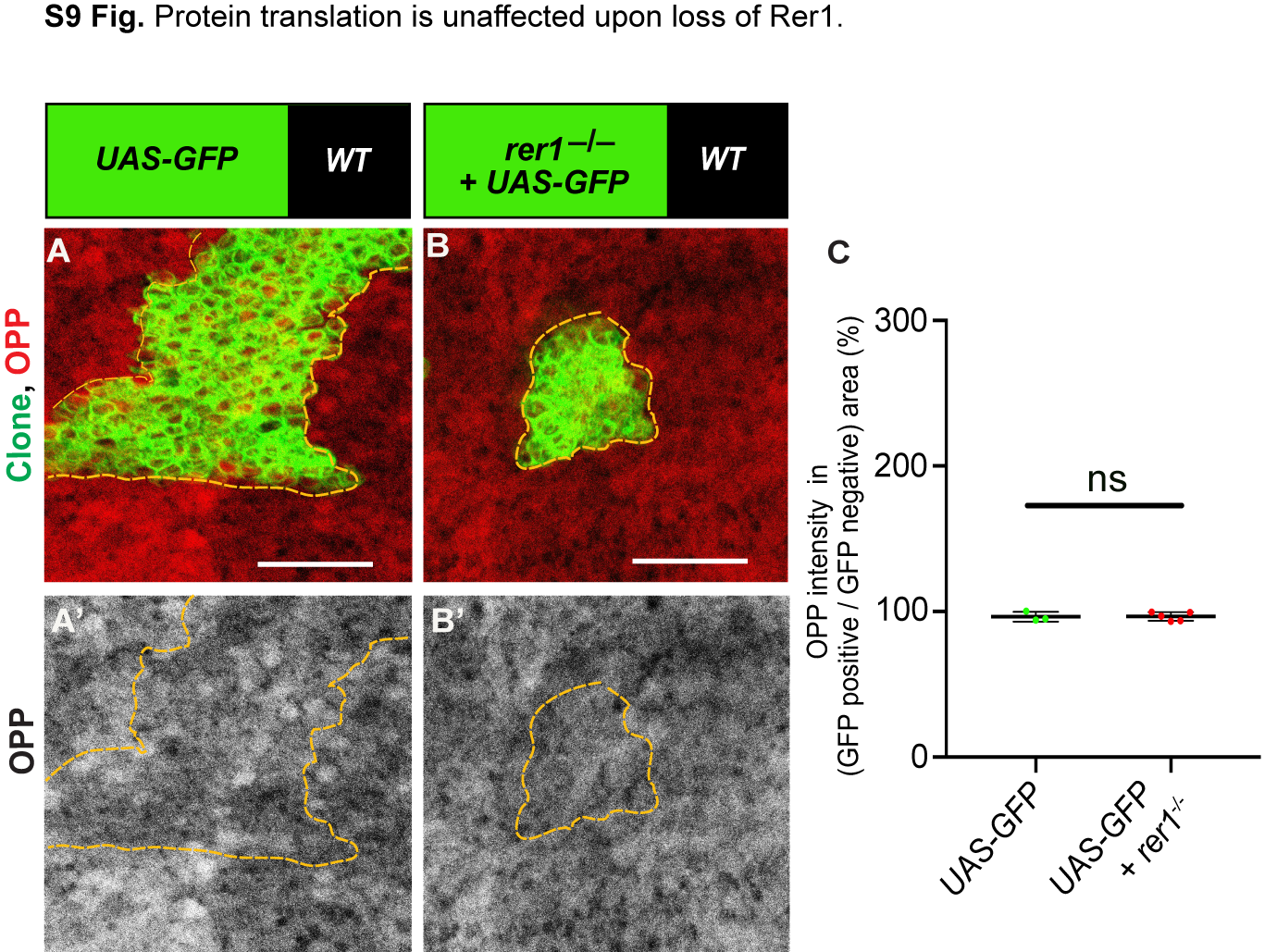

Supplement: S9 Fig — (A-B) OPP assay on third-instar discs containing hs-FLP-induced MARCM clones (96 hrs AHS) of (A) UAS-GFP (control) and (B) UAS-GFP, rer1–/–genotypes. (C) Quantification of the signal intensity of OPP inside the GFP-positive clones with respect to nearby GFP-negative control tissue in UAS-GFP (A; N = 3 wing discs), UAS-GFP, rer1–/–(B; N = 5 wing discs). Borders between the GFP-positive and GFP-negative areas are marked with yellow dotted lines. Statistical analysis in C was performed using the two-tailed Welch’s t-test (p = 0.9431). SB = 20 μm. (TIF) [file pgen.1011171.s009.tif]

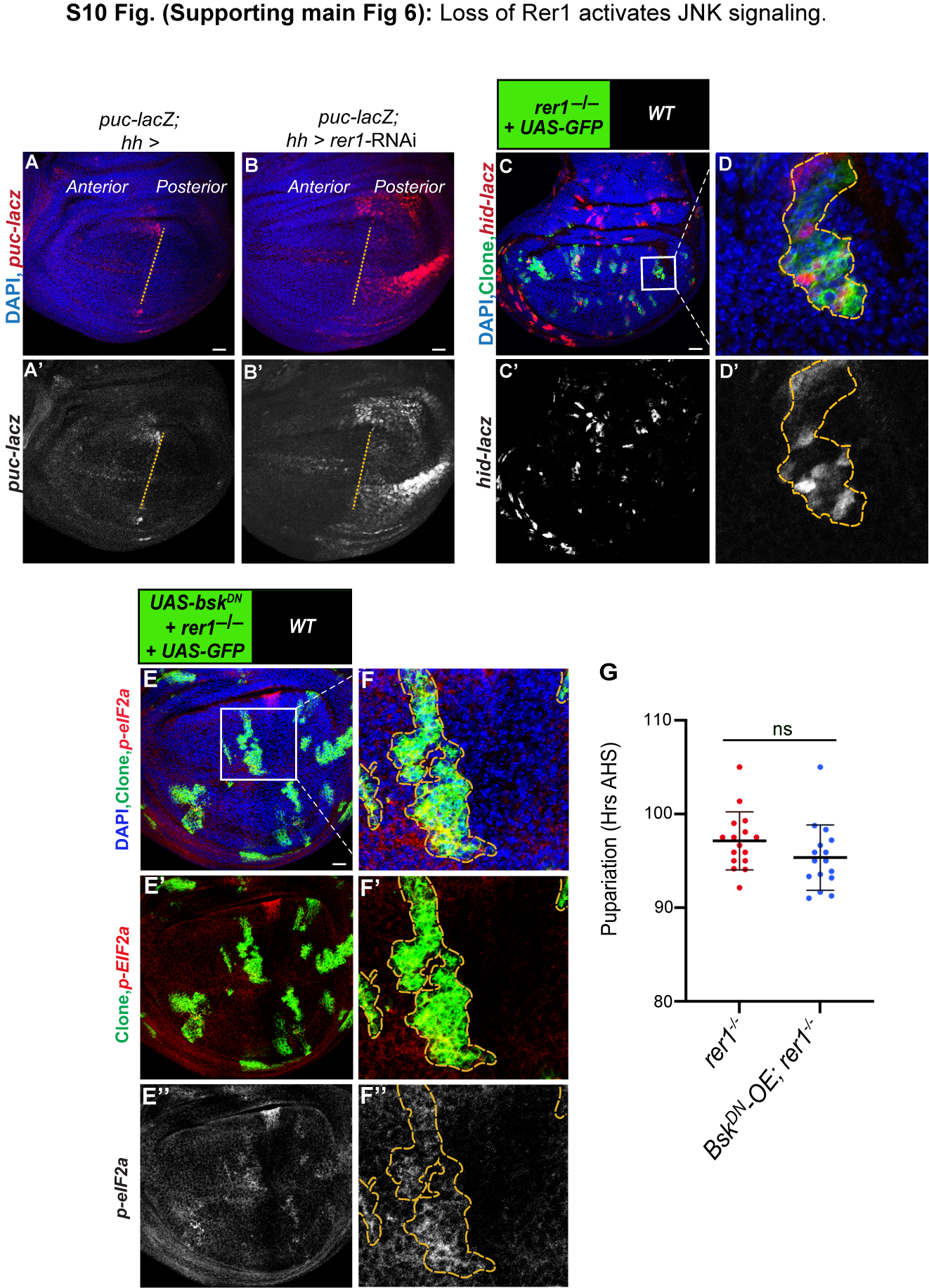

Supplement: S10 Fig — (A-B) Images representing puc-lacZ promoter activities via beta-galactosidase (red) staining on (A-A’) control discs (puc-lacZ; hh-Gal4/+; N = 9 wing discs), or (B-B’) Rer1 depleted discs puc-lacZ; hh-Gal4, UAS-rer1-RNAi; N = 10 wing discs). (C) Third-instar wing discs containing hs-FLP-induced (96 hrs AHS) MARCM clones of UAS-GFP, rer1–/–genotype (N = 4 wing discs), immuno-stained for the beta-galactosidase (red) to mark the hid-lacZ promoter activity. (D-D’) A magnified image of the inset (white box) in C. The interfaces between the clone areas are marked with yellow dotted lines. (E-F) Representative images of hs-FLP-induced MARCM clones (72 hrs AHS) of UAS-GFP, UAS-bskDN; rer1–/–genotype stained with anti-p-eIF2α antibody (N = 6 wing discs). (G) Quantification of pupariation time for UAS-GFP; rer1–/–(N = 16 technical repeats) and UAS-GFP, UAS-bskDN; rer1–/–(N = 16 technical repeats). Statistical analysis in G was performed using the two-tailed Welch’s t-test (p = 0.1389). SB = 20 μm. (TIF) [file pgen.1011171.s010.tif]

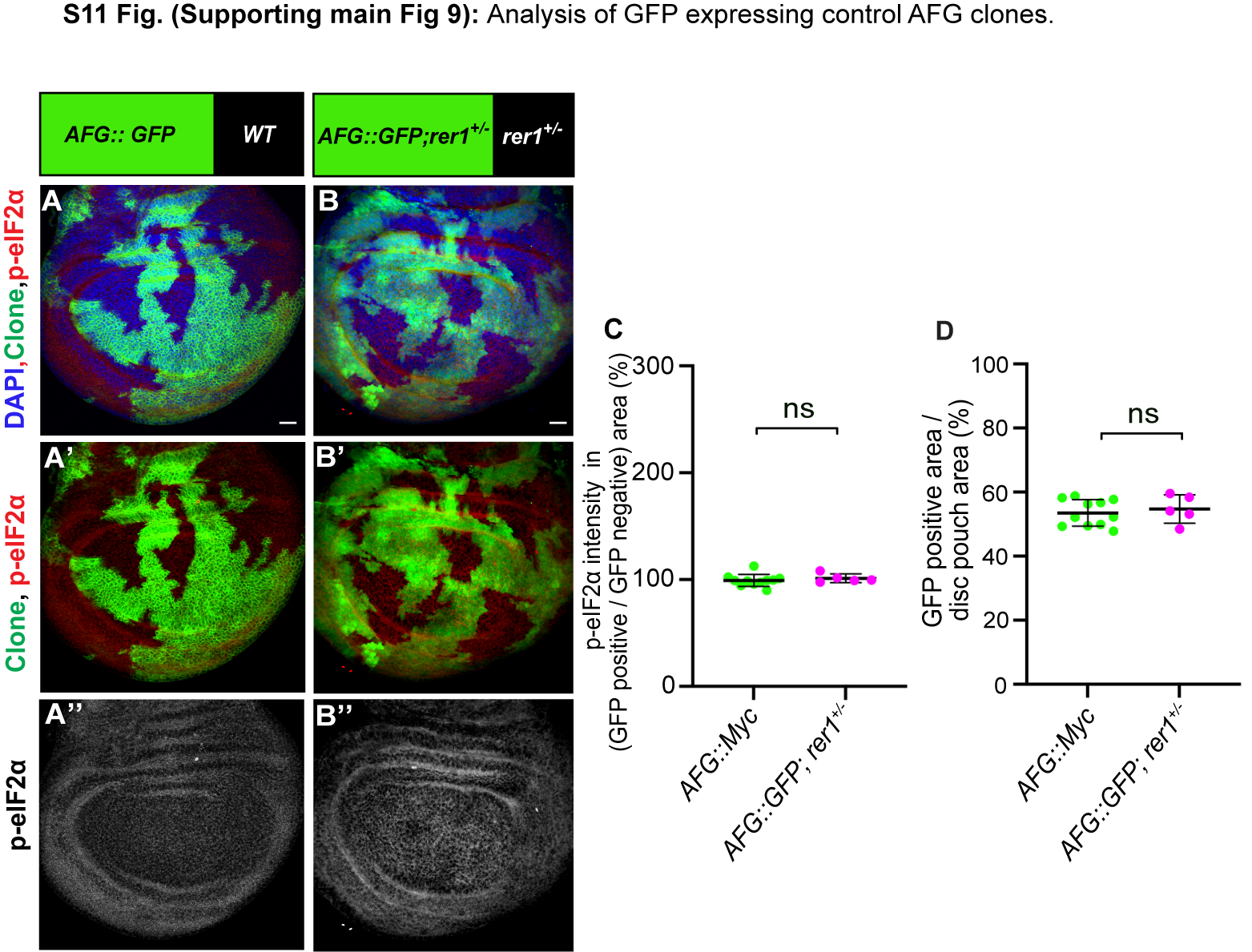

Supplement: S11 Fig — (A-B) Representative images of the wing imaginal discs with hs-FLP induced (48 hrs AEL) Actin-FRT-Stop-FRT-Gal4 (AFG)-control clones overexpressing GFP in either wild-type (A) or rer1+/–background (B), dissected 72 hrs AHS. (C-D) Quantification of p-eIF2α and GFP positive area in AFG:: GFP in WT background (N = 11 wing discs) and AFG:: GFP in rer1+/–background (N = 5 wing discs). Statistical analyses in C and D were performed using the Two-tailed Welch’s unpaired t-test (p = 0.4193 and p = 0.6089, respectively). SB = 20 μm. (TIF) [file pgen.1011171.s011.tif]
