# Supplementary material for: Maintenance of proteostasis by Drosophila Rer1 is essential for competitive cell survival and Myc-driven overgrowth
Source: PLoS Genet. 2024 Feb 26;20(2):e1011171. doi: 10.1371/journal.pgen.1011171 (PMC10919865; doi:10.1371/journal.pgen.1011171)
Supplement: S1 Text — (DOCX) [file pgen.1011171.s012.docx]

**SUPPLEMENTAL EXPERIMENTAL PROCEDURE**

***Drosophila genotypes***

The following genotypes were used in this study:

S1D Fig: ; *GFP-rer1/ UAS-rer1-RNAi; hh-Gal4/+*

S1E-S1E’, S1G-S1G’ Fig: ;; *hh-Gal4/+*

S1F-S1F’, S1H-S1H’ Fig: ; *UAS-rer1-RNAi/+; hh-Gal4/+*

S1K and S1M Fig: *; UAS-GFP/+; hh-Gal4, UAS-GFP/+*

S1L and S1N Fig: *; UAS-GFP/+; UAS-rer1-RNAi, hh-Gal4/+*

S2A Fig: *hs-FLP/ Y; ; FRT82B, Ubi-RFP.nls/ FRT82B, Ubi-GFP.nls*

S2B Fig: *hs-FLP/ Y; ; FRT82B, Ubi-RFP.nls/ neoFRT82B, ry^506^, rer1^KO^*

S2C Fig: *hs-FLP/ +; ; FRT82B, Ubi-RFP.nls/ FRT82B, Ubi-GFP.nls*

S2D Fig: *hs-FLP/ +; ; FRT82B, Ubi-RFP.nls/ neoFRT82B, ry^506^, rer1^KO^*

S2F-S2F’, S2G-S2G’ Fig: *hs-FLP/+; ; FRT82B, Ubi-RFP.nls/ neoFRT82B, ry^506^, rer1^KO^*

S3A-S3A’’,S3B-S3B’’ Fig: *hs-FLP, UAS-GFP/+; UAS-rer1-RNAi/+ ; tubP-Gal4, neoFRT82B, tubP-Gal80/ neoFRT82B, Ubi-mRFP.nls*

S4A-S4A’’’, S4B-S4B’’’ Fig: *; GFP-rer1/+;*

S5A-S5A’ Fig: ;; *hh-Gal4, UAS-GFP/+*

S5B-S5B’ Fig: ; *UAS-rer1-RNAi /+; hh-Gal4, UAS-GFP/+*

S5C-S5C’ Fig: ; *+/+; hh-Gal4, UAS-GFP/UAS-rer1-RNAi*

S5D-S5D’ Fig: ; *+/+; hh-Gal4, UAS-GFP/UAS-rer1-RNAi*

S5E-S5E’’, S5F-S5F’’ Fig: *hs-FLP, UAS-GFP/+; UAS-rer1-RNAi/+; tubP-Gal4, neoFRT82B, tubP-Gal80/ neoFRT82B, Ubi-mRFP.nls*

S6A-S6A’’, S6B-S6B’’ Fig: ;;*hh-Gal4/UAS-rer1-RNAi*

S6C-S6C’’, S6D-S6D’’ Fig: ;;*hh-Gal4/+*

S7A-S7A’ Fig*: ;UAS GFP/+; hh-Gal4, UAS-GFP/+*

S7B-S7B’ Fig: *;UAS-PERK-RNAi /+; hh-Gal4, UAS-GFP/+*

S7C-S7C’ Fig: *;UAS-GCN2-RNAi /+; hh-Gal4, UAS-GFP/+*

S7D-S7D’ Fig: *;UAS-GFP/+; UAS-rer1-RNAi, hh-Gal4/+*

S7E-S7E’ Fig: *;UAS-PERK-RNAi /+; UAS-rer1-RNAi, hh-Gal4/+*

S7F-S7F’ Fig: *;UAS-GCN2-RNAi /+; UAS-rer1-RNAi, hh-Gal4/+*

S8A-S8A’, S8B-S8B’ Fig: *; UAS-GADD34/+; UAS-rer1-RNAi, hh-Gal4 /+*

S9A-S9A’ Fig: *hs-FLP, UAS-GFP/+;; tubP-Gal4, neoFRT82B, tubP-Gal80/ neoFRT82B, Ubi-mRFP.nls*

S9B-S9B’ Fig: *hs-FLP, UAS-GFP/+;; tubP-Gal4, neoFRT82B, tubP-Gal80/ neoFRT82B, ry^506^, rer1^KO^*

S10A-S10A’ Fig: *+/+; +/+; hh-GAL4/ pucE69-lacZ*

S10B-S10B’ Fig: *+/+; +/+; hh-GAL4, UAS-rer1-RNAi / pucE69-lacZ*

S10C-S10C’, S10D-S10D’ Fig: *hs-FLP, UAS-GFP/+; hid-lacz/+; tubP-Gal4, neoFRT82B, tubP-Gal80/ neoFRT82B, ry^506^, rer1^KO^*

S10E-S10E’’, S10F-S10F’’: *hs-FLP, UAS-GFP/UAS-bsk^DN^; +/+; tubP-Gal4, neoFRT82B, tubP-Gal80/ neoFRT82B, ry^506^, rer1^KO^*

*S11A-S11A’’: AFG/hs-FLP; +/+; UAS-GFP/+*

*S11B-S11B’’: AFG/hs-FLP; +/+; UAS-GFP/neoFRT82B, ry^506^, rer1^KO^*

**Acridine orange staining**

Acridine orange staining was performed by dissecting the third instar larval wing imaginal discs in PBS (1X) followed by 2 minutes of incubation in 0.6 mg/ml acridine orange + PBS (1X) solution [[1]](https://paperpile.com/c/5HwMxC/N5anp). Afterwards, wing discs were rinsed very briefly in PBS (1X) before mounting.

**OPP assay**

For the detection of nascent protein synthesis, Click-iT^TM^ Plus OPP Alexa Fluor^TM^ 647 Protein Synthesis Assay Kit (Thermo Fisher Scientific) was used, and the procedure was followed as described:

Third instar larvae (4-5) were dissected in Schneider′s Insect Medium (SM) and proceeded with only wing imaginal discs attached to the head complex. The samples were transferred into SM containing component-A (1:1000) and incubated for 15 min at room temperature (RT). The solution containing component-A was removed, and the samples were fixed in 4% PFA for 30 min. Thereafter, PBT and PBS washes were given to add the reaction cocktail. After the 30 min of incubation, the reaction cocktail was removed and washed carefully with component F. The component F was removed, rinsed the sample with PBS, and component G (1:1000) was added to stain the nucleus. Wing discs were mounted in Vectashield mounting medium (Vector laboratories) on glass slides (1mm BlueStar micro slides) and scanned quickly afterwards. Staining and microscopy conditions for samples used were identical.

**ROS detection (DHE uptake assay)**

DHE (Dihydroethidium) uptake assay was performed as per the protocol mentioned in ​​Owusu-Ansah et al., 2008 [[2]](https://paperpile.com/c/5HwMxC/XS4p). In brief, the wing imaginal discs from the third-instar larvae, expressing *rer1*-RNAi GD 23204 with *hh-Gal4*, were dissected in the Schneider’s medium, at room temperature (25°C). Wing discs were then incubated in 30uM DHE (Invitrogen) reconstituted in the Schneider’s medium, for 5 minutes, on an orbital shaker at RT. Subsequently, the tissue was washed thrice with the Schneider’s medium, fixed in 7% formaldehyde in 1X PBS solution for 5 minutes. Furthermore, wing discs were stained with DAPI for 5 min, mounted in Vectashield mounting medium (Vector laboratories) and imaged immediately with a confocal microscope.

**Adult wing size measurement**

The area of the individual adult wing from males and females, was measured along the edge (dotted lines shown in S1K-S1N and S2A-S2D Figs) using ImageJ software.

***Reference***

1. [Robbins E, Marcus PI. DYNAMICS OF ACRIDINE ORANGE-CELL INTERACTION. I. INTERRELATIONSHIPS OF ACRIDINE ORANGE PARTICLES AND CYTOPLASMIC REDDENING. J Cell Biol. 1963;18: 237–250.](http://paperpile.com/b/5HwMxC/N5anp)

2. [Owusu-Ansah E, Yavari A, Banerjee U. A protocol for in vivo detection of reactive oxygen species. Protoc Exch. 2008. doi:](http://paperpile.com/b/5HwMxC/XS4p)[10.1038/nprot.2008.23](http://dx.doi.org/10.1038/nprot.2008.23)
