# Supplementary material for: Maintenance of proteostasis by Drosophila Rer1 is essential for competitive cell survival and Myc-driven overgrowth
Source: PLoS Genet. 2024 Feb 26;20(2):e1011171. doi: 10.1371/journal.pgen.1011171 (PMC10919865; doi:10.1371/journal.pgen.1011171)
Supplement: Attachment — Submitted filename: Paul et al Responses to Reviewers PlosGen.pdf [file pgen.1011171.s014.pdf]

Response to Reviewers' Comments

PGENETICS-D-23-00557R1

We would like to thank all the reviewers for the comments, which led to significantly improved manuscript. Below we provide a point-by-point response to the comments.

Comments to the Authors:

Reviewer #1:

Paul et al present a manuscript on the role of Rer1 role in protein homeostasis and Myc-driven super-competition. Although the manuscript compiles interesting data, the study has limitations that make it difficult to recommend for publication. There are three important results. First, Rer1 is an essential gene in Drosophila. Second, Rer1 mutant cells are eliminated from wing epithelium via cell competition. Lastly, Rer1 is required for Myc-driven cell competition. Currently, an in-depth study of any of the three points is missing. Addressing the below-mentioned points would help authors to improve their manuscript:

1. As per the title of the manuscript, the major claim of the authors is that Rer1 is required for the maintenance of protein homeostasis. The supporting evidence is that Rer1 localizes in Golgi & ER and loss of Rer1 results in increased p-eIf2α levels. A more detailed study is required here to dissect if Rer1 has a direct role in the regulation of protein homeostasis or if p-eif2α levels increase because of activation of proteotoxic stress signaling in Rer1 mutant, as shown recently in ribosome heterozygous cells or mahjong mutant cells (Langton et al 2021 PLoS genetics).

Moreover, does the Rer1 mutant accumulate unfolded proteins? Also, does PERK or GCN2 kinase activity go up or does the dephosphorylation of p-eIf2alpha go down? Perturbation of protein homeostasis also affects autophagic and proteasomal flux, addressing all these points will make the study more comprehensive.

> We thank Reviewer #1 for pointing out mechanistic weaknesses and suggesting additional experiments. We have now analyzed the effects of Rer1 depletion on proteotoxic stress and accumulation of unfolded proteins using the Proteostat assay. We find that the depletion of Rer1 causes mild accumulation of unfolded protein (new Figure 7E). The data is provided along with Myc-overexpression in Rer1 depleted cells, where we observed a strong enhancement in protein aggregation (new Figure 7G), suggesting that Rer1 is required for protein homeostasis.

To further dissect the mechanisms of p-eIF2α induction, we downregulated either PERK or GCN2 in *rer1* mutant cells. The data in new Figure 4A-E shows that depletion of PERK, and not GCN2, caused downregulation of p-eIF2α levels in cells lacking Rer1, suggesting that loss of Rer1 leads to PERK-mediated phosphorylation of eIF2α, providing additional support to the role of Rer1 in proteostasis. Of note, these results also provide a solid basis for the in-depth exploration of downstream impacts of UPR activation, for instance related to autophagy and/or proteasomal flux and which are beyond the scope of the current study.

2. *Regarding the role of Rer1 in cell competition, the data presented in Fig 2 A-E does not support that Rer1 has a role in cell competition. This data could also be possible because of protein perdurance in somatic clones and mutant cells display cell autonomous cell death*.

> The data in figure 2A-E showed that the *rer1* mutant clones fail to survive in the wing epithelium. We agree that this data alone is not sufficient to conclude that the *rer1* mutant clones are eliminated via cell competition. Accordingly, we have now moved the data to Figure 1B-E. Our conclusion that the *rer1* mutant cells are eliminated via cell competition is supported by the following observations: 1) the *rer1* mutant clones, as well as *rer1*-RNAi expressing clones (new S3 Fig), show cell death preferentially at the boundary with the normal cells. 2) the *rer1* mutant clones perform better when surrounded by the *Rps3+/-* cells and more importantly the cell death is now observed in the *Rps3+/-* cells and significantly reduced in the *rer1* mutant cells (new Figure 2A-F).

3. The current model for competitive elimination of RpS3 heterozygous is through Xrp1. Xrp1 regulates proteotoxic stress signaling in RpS3 cells (Langton et al 2021, PLoS Genetics). Thus, data related to the continuous elimination of RpS3/+ cells in the presence of Rer1 mutant, as shown in Fig 2F-I, shows that most likely Xrp1 is still activated in Rp3/+ cells. Interestingly, Xrp1 is known to be active in RpS3/+ cells in a cell-autonomous manner (Lee et al 2018, Dev cell). Therefore, how this data supports the role of Rer1 in cell competition is not clear.

> We would like to point out that the primary aim of the experiment analyzing the competition between *RpS3+/-* (which are also *rer1+/-)* and *rer1-/-* cells was to analyze cell autonomous death in *rer1-/-* clones. It is indeed interesting to note that, while both *RpS3+/-* and *rer1-/-* cells are eliminated due to proteotoxic stress, *rer1-/-* cells performed better and showed reduced cell death, when abutting *RpS3+/-*. Several potential reasons, including differential activity of Xrp1, can be the cause of this change in the fate of *rer1-/-* mutant cells from loser to winner. We have now included these points in our updated discussion.

4. The data presented in Fig 1B-E does support the role of Rer1 in cell competition. However, this data need to be validated by knocking down Rer1 through its RNAi and then showing boundary cell death and clone area compared to control clones.

> We thank the reviewer for this excellent suggestion. We have now analyzed cell death in the *rer1*-RNAi MARCM clones. Consistent with the *rer1* mutant clone, we observed higher Dcp-1 levels in cells at the boundary of *rer1*-RNAi expressing clones (new S3B figure). Moreover, these clones also showed reduced growth (new S3D Fig) and higher p-eIf2α (S5E-F Fig).

5. The model suggested for Rer1 cell competition would be more complete by studying genetic epistasis between p-eIf2α and JNK signaling in Rer1 mutant cells.

> Thank you for the suggestion. We have now performed the epistasis experiment by analysing the effect of blocking JNK signaling on p-eIf2α levels in *rer1* mutant cells. We find that the expression of DN-Basket, which suppresses JNK signaling, did not affect p-eIf2α levels in *rer1* mutant cells (new S10E-F Fig); however, it could partially rescue growth of *rer1* mutant clones

(new figure 6G). These results suggest that JNK signaling was activated downstream of p-eIf2α, restricting the growth of *rer1* mutant cells.

6. p-eIf2α role in cell competition is already known (Naotaka Ochi et al 2021 PLoS Genetics) and it plays a cytoprotective role in different stress conditions. Therefore, how its upregulation results in the elimination of Rer1 is not clear. Moreover, it would be interesting to examine if GADD34 overexpression rescues cell autonomous cell death that occurs upon knockdown of Rer1 in the posterior compartment. This will help to dissect if p-eIf2α has a cell protective role or has a role only in cell competition.

> As suggested, we have analyzed the effect of blocking GADD34 on the cell death due to loss of Rer1. We now show that the expression of GADD34 in the posterior compartment rescued cell death caused by Rer1 depletion (new S8B Figure). Furthermore, we find that expression of GADD34 in *rer1* mutant clones showed a reduction in DCP-1 levels and rescue of growth (Figure 5E and quantified in G). Moreover, and consistent with GADD34 overexpression, depletion of PERK restored growth of *rer1* mutants and rescued cell death (new Figure 4F). These results show that the elimination of cells lacking Rer1 is mediated by the phosphorylation of eIF2α levels.

We propose that Rer1 itself could be part of the cytoprotective processes; therefore, loss of Rer1 would make the cytoprotective role of p-eIF2α less effective. This is supported by the Myc-overexpression experiments, where we observe an increase in protein aggregation upon loss of Rer1 (new Fig7D-H). Moreover, cells show more stress when Myc is overexpressed in the *rer1* heterozygous background as compared to the control (new Figure 9A-C).

7(a). The proposed role of Rer1 in Myc cell competition needs further investigation. First, the way this manuscript is written looks like proteotoxic stress activation is associated with Myc super-competition. However, this is not the case. Proteotoxic stress activation is shown to be required for Myc overgrowth phenotype and any association with its super-competition is not known. Therefore, it is important to show that activation of proteotoxic stress activation is required for Myc super-competition.

Additionally. first paper in which the role of Myc was demonstrated in super-competition, it was also shown that differential growth is not sufficient for cell competition (de la Cova, 2004, cell). Therefore, any conclusion on Myc super-competition based on only the growth behavior of cells would lead to the wrong conclusion as shown in Fig 6 of this manuscript.

> We thank the reviewer for pointing out this error in our conclusions. Indeed, the UPR has been shown to promote Myc-induced overgrowth and has not been directly linked to super-competition. We have rewritten this part and modified our conclusions to better define the role Rer1 in Myc-induced overgrowth. Further, we updated our model to highlight the cytoprotective function that Rer1 plays in Myc-overexpressing cells (see also response to 6).

7(b). Moreover, if proteotoxic stress activation has a protective role in Myc overexpressing cells (Nagy et al 2013 PLoS Genetics), then why do these cells with simultaneous loss of Rer1 have

smaller clone size? Is it possible that higher p-eIF2alpha levels shown by these cells are because of additive regulation of p-eIF2aplha levels by Myc overexpression and Rer1 loss?

> We have addressed this issue with additional experiments. Since our data showed that Myc overexpression increased the levels of Rer1, we tested if only lowering the levels of Rer1 in the animal, rather than a complete loss of function in clones, would modulate Myc-induced growth. Thus, we generated Actin-flip-out Gal4 clones overexpressing Myc in either wildtype or *rer1+/-* heterozygous flies. We observed that Myc-overexpressing clones showed higher levels of stress and reduced growth in *rer1+/-* background as compared to the control (new Figure 9A-D). These results show that higher levels of Rer1 are required for mitigating stress upon Myc-overexpression and thereby supporting overgrowth.

8. What is the significance of Rer-1 upregulation in Myc overexpressing cells? Does Rer1 overexpression result in Myc overexpressing cells grow even better and display lower proteotoxic stress signaling.

> Our results suggest that Myc-overexpression led to an increase in Rer1 levels, which could be a feedback response to mitigate proteotoxic stress upon Myc-overexpression. To test the importance of high Rer1 levels, we have performed new experiments where we observed that Myc-driven proteotoxic stress was further enhanced in *rer1+/-* flies, with a concomitant reduction in the overgrowth (new Fig 9A-C), supporting the dependency of Myc on higher level of Rer1.

Nevertheless, we attempted to test whether further increase in Rer1 levels could provide additional advantage to Myc-overexpressing cells, by generating a UAS-*rer1* for overexpression. However, we find the overexpression of Rer1 using various Gal4 drivers (for example, *ptc-Gal4* [wing discs] or *ubx-Flp* mediated clonal expression of Rer1) led to a strong increase in cell death (Figure below). It is likely that this toxicity is due to a strong overexpression of Rer1. Therefore, we believe that the Gal4-mediated co-overexpression of Rer1 and Myc, requiring a precise control of expression levels, may not be a feasible experiment to test for overgrowth. Alternative tools will be required for an independent controlled expression of Rer1 and Myc, which is beyond the scope of the current study.

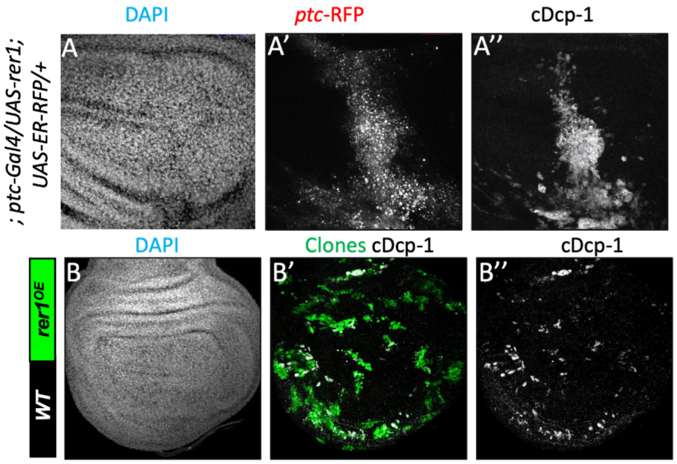

Figure: Overexpression of Rer1 leads to autonomous cell death. Dcp-1 staining on wing discs expressing Rer1 with either *ptc-Gal4* (A) or *ubx-Flp* mediated MARCM clones.

Minor points:
1. Abstract "Cell competition is a developmental phenomenon that allows the selection of healthier cells in a developing tissue"
In the above-mentioned line in the abstract, authors have suggested cell competition as a developmental phenomenon. However, this is not the case, and it has much broader significance (Neerven et al 2022, Nature Reviews Molecular Cell Biology).

> Thank you for the comment. We have edited our abstract and modified these sentences.

Reviewer #2:

Cell Competition is key mechanism utilised to eliminate potentially dangerous cells for the living organism. In the last decade, the physiological relevance of this phenomenon has been discovered as well as players involved in the elimination of unfit cells. However, the mechanisms which induce unfit cells remain still largely unknown. Paul and Co-authors present in this article a novel Cell Competition regulator Rer1 which appears also to be required for supercompetition. The manuscript is well written and shows convincing evidence that Rer1 regulates cell fitness in wt and supercompetition. Furthermore, I believe that the manuscript will be of interest for the general audience of the journal.

However some points should be clarified or further develop:

Major points:
1- Rer1 downregulation in non-competitive scenarios is sufficient to trigger cell death (Figure S1). How authors reconcile this fact with the role Rer1 in cell competition?

> To test if the expression of *rer1*-RNAi caused cell-autonomous death, we generated MARCM clones expressing *rer1*-RNAi. We observed that similar to the results obtained with *rer1* mutants, depletion of *rer1* in clones showed higher boundary cell death (new S3 Fig). Furthermore, we have observed that complete loss of Rer1 (in mutant clones) did not show cell autonomous death (new Fig 2). Therefore, we believe that observation of cell death in the posterior compartment might be due to non-uniform expression of Gal4 or differential RNAi-mediated depletion of Rer1.

2- Authors should test the activation of JNK pathway in competitive settings if they want to make statements about the downstream signalling (Fig.3). Given the challenging genetics, authors may want to use in this case Anti-ACTIVE® JNK to detect the activation of JNK pathway in the rer1-/- clones.

> Thank you for this excellent suggestion. We have now analyzed the JNK activity in the *rer1* mutant clones. However, instead of the commercially available Anti-ACTIVE® JNK antibodies, which we find to be of low sensitivity, we used a well-established JNK specific reporter, TRE-DsRed (Chatterjee and Bohmann, 2012). As shown in new Figure 6A-B, we observed an increase in the levels of TRE-DsRed in clones. Furthermore, as JNK inactivation showed mild rescue in the growth of *rer1* clones, we have modified our conclusions to suggest that JNK

signaling partially restricts the growth of *rer1* clones, consistent with the observation in Rp+/- clones (Kucinski et al., 2017).

3- Authors show cell competition phenotypes when downregulating or mutating rer1. It would be very interesting to analyze if Rer1 overexpression in clones is sufficient to induce supercompetitor cells in a wt background. Similarly, to test whether Rer1 overexpression in combination with Myc overexpression in clones, increases the competitive behavior of the Myc overexpressing clones alone.

> Encouraged by this comment (also suggested by Reviewer#1), we have now analyzed cell death upon overexpression of Rer1 using either *ptc-Gal4* or *ubx-flp-induced* MARCM clones. In both conditions, we observed a strong increase in the Dcp-1 levels in the Rer1 overexpressing cells. It is likely that this toxicity is due to very high overexpression of Rer1. Therefore, we believe that the Gal4-mediated co-overexpression of Rer1 and Myc, requiring a precise control of expression levels, may not be a feasible experiment to test for overgrowth. Alternative tools will be required for an independent controlled expression of Rer1 and Myc, which is beyond the scope of the current study.

Minor points:

1- Figure 1B does not show the merge of RFP and DCP1, it is just showing the RFP.

> Apologize for the mistake. We have merged the DCP1 channel along with the RFP channel in the updated image (Figure 1F-F'').

2- Is Figure 4A showing the experiment 96h AHS? Clones from Figure 1B-D (96h AHS) and Figure 2C (96h AHS) look much bigger.

We apologize for this error. The images were of 48 hrs clones. We have now updated the figure (new Fig 3) by adding the 96 hrs AHS *rer1* mutant clones and rescue.

3- Fig. 6: Authors only demonstrate the requirement of Rer1 in supercompetition they do not show higher levels of Rer1 in competitive settings (i.e. Supercompetition).

> Our results have shown that overexpression of Myc leads to increase in Rer1 levels (Fig 7 A-B). To further strengthen our conclusions, we performed Myc-overexpression on *rer1+/-* flies, which showed further enhancement in proteotoxic stress in Myc-overexpressing cells and restricted the overgrowth (Fig 9). We believe these results provide better evidence to functionally link the importance of higher Rer1 levels in Myc-driven overgrowth.

4- Fig. 6J, Distance is not measured in a.u.. Authors may want to say microns/pixels.

> As suggested, we have edited the unit of distance to microns.

5- Figure legends for some panels are missing, eg: Fig.1 E".

> We apologize for the mistake. Legends have been corrected in the updated figures.

6- Clarify what authors refer for the sample size (N), number of discs or number of clones.

> We thank you for pointing out this oversight. We have now clarified the 'N' numbers in the respective figure legends.

Reviewer #3:

In this work Paul et al generated a rer1 null allele, which presented early larval lethality. They showed that loss of Rer1 leads to increased proteotoxic stress and JNK activation. Additionally they present evidence that Rer1 mutant clones are eliminated through cell competition. They showed that Rer1 levels are upregulated upon Myc overexpression, by using a GFP tagged rer1 genomic rescue construct. The support that Rer1 has a cytoprotective role in Myc induced proteotoxic stress, which is essential for supercompetition. Rer1 acts as a novel regulator of protein homeostasis in Drosophila and reveal its role in competitive cell survival.

Strong points: The author by generating a new Rer1 knockout allele, they manage to show that Rer1 indeed is an essential factor in Drosophila as it is in other organisms and that Rer1 mutant cells presents characteristics of loser fate. This works comes to add another example of proteotoxic driven cell competition.

Weak point: The authors aim to demonstrate that Rer1 has a protective role in Myc induced proteotoxic stress, however, the data does not fully support that the effect of Rer1 is specific to Myc induced mechanism or it is due to its essential function in the cell.

Major Revisions

1. Please provide Dcp1 staining in the experiment with the partial rescue of clonal size of rer1-/- cells by overexpression of bskDN, to show a direct effect on the cell competition hallmark, Dcp1. This is important since there is difference in the size of rer1-/- clone in Figure 2 according to time after heat shock. Therefore, any developmental delay or difference in egg deposition could have an impact in the clone size, not necessarily the competition between the clones and the background cells.

> Thank you for this constructive criticism. We have repeated the experiment to analyse the Dcp-1 levels in the *rer1* mutant clones expressing bskDN. However, in line with the previous observations on the effect of blocking JNK in Rp+/- cells (Kucinski et al., 2017), we did not observe any significant difference in Dcp-1 levels. It is possible that JNK limits growth of the *rer1-/-* (and Rp+/-) cell via an alternate mechanism.

These results prompted us to reassess if the effect of bskDN on the *rer1* mutant clone size is due to developmental delay. Thus we performed the experiment to reanalyze the clone size along with the pupariation time. In these experiments, we did not observe any difference in the pupariation time between flies with *rer1* mutant clones and *rer1* mutant clones expressing bskDN (S10G figure). However, consistently we observed a rescue in the clone size. We conclude that JNK plays a partial role in the restricting growth of *rer1*-/- cells.

2. Could the authors show reduced Dcp1 for the experiment that they rescue clone size by overexpressing GADD34? To exclude that the clone difference is an secondary effect of developmental timing and not due to competition. (logic similar with the rescue by BskDN).

> We have performed the Dcp-1 staining on GADD34 overexpression in either *rer1* mutant clones (New figure 5D, and quantified in G) or co-expression with *rer1*-RNAi (S8 Figure B). In both conditions we observed significant reduction in Dcp-1 levels.

In addition, we also analyzed the activation of UPR and show that loss of Rer1 leads to PERK-mediated phosphorylation of eif2α and downregulation of PERK in *rer1* mutants showed rescue of clone size and reduction in Dcp-1 levels (New Figure 4), similar to GADD34 overexpression. On the other hand, downregulation of Gcn2 in *rer1* mutant clones neither showed any rescue effect on p-eif2α levels nor rescued *rer1* mutant clones (New Figure 4D, J). Together with these results we conclude that phosphorylation of eIF2α caused the elimination of *rer1* mutant cells.

3. The authors in order to explore if Rer1 plays a role in the growth of Myc-overexpressing cells, they state that "We generated Myc-overexpressing clones in the wing disc, in either wild-type or rer1–/– background". According to their genotype (Fig 6C : hs-FLP, UAS-GFP/+; UAS-Myc/+; tubP-Gal4, neoFRT82B, tubP-Gal80/ neoFRT 82B, ry506, rer1KO) , the background is rer1+/- heterozygous and only the myc overexpressing clones will be rer1-/-.

> We apologize for the error and have now corrected this statement to "Thus we induced MARCM clones overexpressing Myc in either control or *rer1*$^{-/-}$ clones" (Page 10).

4. They showed in Figure 6, that the overgrowth phenotype observed due to the overexpression of Myc was reduced in the rer1–/– cells (Fig 6A – D; quantified in 6E, compare 6B and 6C), underscoring that Rer1 is required for Myc-induced overproliferation. Earlier in the manuscript authors have solid data that Rer1 is an essential protein even in wild type cells. How the authors can exclude the possibility that the absence of rer1 reduces Myc-induced overproliferation, not due to a specific effect on the Myc mechanism, but independently as an essential factor. Therefore, that would mean that Rer1 is required in the cells independently of the Myc induced mechanism.

> Thank you for the comment. Kindly refer to our answer to the similar concerns of Reviewer#1 (point 7b).

5. What happens when the authors have the extra copy of the rer1 locus (GFP tagged). Can they see if the supercompetitor status of myc overexpressing cells is increased more with extra Rer1 protein, by checking the clone size for example, to support the cytoprotective role of Rer1 in Myc cells?

> Unfortunately, we could not perform the experiment to analyze Myc-driven overgrowth in flies with an extra copy of the *rer1* gene (*GFP-rer1*). However, we believe that our results showing suppression of Myc-induced overgrowth and enhancement of Myc-driven stress in *rer1+/-* flies (Figure 9A-D, kindly also see our response to point 7b of Reviewer#1) support our conclusions that higher levels of Rer1 provide cytoprotection to the Myc-overexpressing cells, supporting overgrowth. Additionally, we also observed enhancement in protein aggregation upon depletion of Rer1 in Myc-overexpressing cells (Figure 7D-H), further supporting our conclusions.

6. What happens if they have heterozygosity or homozygosity of rer1 locus in all cells. For

example, would it be easy to perform the experiment using this genotype: hs-FLP, UAS-GFP/+; UAS-Myc/+; tubP-Gal4, neoFRT82B, tubP-Gal80, rer1KO / neoFRT 82B, ry506, rer1KO. In that case all the cells will lack rer1, not only the myc overexpressing clones.

> Thank you for this suggestion. The mentioned experiment would have been very interesting to investigate, but this final genotype will not allow the animal to survive as *rer1-/-* flies are lethal. Therefore we could not perform the suggested experiment.

7. The above additive effect could also explain the higher levels of p-eIF2a when cells overexpress Myc but lack Rer1 protein. Someone could support that the cells have two different independent stressors that increases p-eIF2a levels and reduces growth of the clones. I think it is important to strengthen this conclusion by other approaches.

>Thank you for this comment. Kindly refer to our answer to the similar concerns of Reviewer#3, comment 4.

Minor Revisions

1. In the abstract where they mention ER add also endoplasmic reticulum (ER)

>As suggested, endoplasmic reticulum (ER) is added in the abstract.

2. Authors mention that: "Previous studies have suggested that the loser fate of Rp+/- cells is due to a reduction in protein translation [7–9]". Actually, Lee et al 2018 (citation #7) suggested that reduced translation was likely responsible for the slow growth of Rp+/- cells, but they did not proposed that loser fate of Rp+/- cells is due to a reduction in protein translation.

>We have modified the statement in the updated manuscript (first paragraph of introduction) as "Earlier studies suggested that the loser fate of slow growing $Rp^{+/-}$ cells possibly results from reduced protein translation (Kale et al., 2018; Lee et al., 2018; Moreno and Basler, 2004)". We would like to thank the reviewer for mentioning this.

3. Authors mention that: "However, recently it was shown that it is a result of increased proteotoxic stress due to protein aggregation [10–13]." The word "shown" will be misleading for the audience. I think it is better to use a word not so loud since the existed data do not clearly support that loser fate of Rp+/- cells in Drosophila is due to proteotoxic stress due to protein aggregation. There is still inconsistency in the field.

> Thank you for this suggestion. We have modified these statements in the introduction (first paragraph) to include other reasons for the proteotoxic stress in *Rp+/-* cells, such as, Xrp-1, leading to their elimination.

4. In Figure S1 panel I, the Y axis has the "%" symbol. Do the authors mean that the ratio of [Anterior Dcp1]/ [posterior Dcp1] is 32%? Does this mean that posterior compartment has ~3 times more Dcp1 compared to anterior in hhGal4::UAS-rer1-RNAi experiment? In Figure S1F it looks quite more Dcp1 in posterior vs anterior, not just 3 fold. Also, the control hh-Gal4 in panel I should be 100, since the Dcp1 does not differ between anterior and posterior compartment.

Therefore the ratio is 1 and if we are transforming this to %, must be 100%. Same comments for panel J.

> Thank you for the suggestion. We corrected this now (S1 Figure I and J), and quantified as Number of cDcp-1 punctae in posterior compartment/anterior compartment in the control (*hh*>) disc and *rer1* knockdown (*hh*> *rer1*-Ri) disc. Apologies for the error in the quantification.

5. In panel C of Figure 1, the rer1-/- clone that is located in the ventral side of the posterior compartment does not present competitive cell death at the boundaries. Contrary the twin spot (rer1+/+) shows boundary cell death. Have the author noticed any difference in rer1 clones and twin spots between ventral and dorsal compartments? Maybe they could provide more examples of clones.

> Thank you for the comment. We have analyzed more examples of *rer1-/-* clones, however we did not observe any noticeable difference in the cell death in the dorsal and ventral compartment. We have now added another example of Dcp-1 in 72 hrs *rer1-/-* mitotic clone (S2F-G), where cell death can be observed in both compartments. Furthermore, similar cell death can be observed in *rer1-/-* mitotic clone found in dorsal and ventral compartments (Fig 4H and 6E).

> In this experiment (Figure 1C-D) they mention that they have generated clones 96hs after heat shock. Have the authors done Dcp1 staining in 72 hours clones, where the rer1-/- clones are bigger and in the process of elimination (according to their data in Figure 2B)?

> A wing disc harboring *rer1* mutant clones (72 hours AHS) is added in the S2 Figure F-G. The inset in F showed magnified clones depicting cell death (blue arrow) in the RFP negative *rer1* mutant tissue in G.

6. In panel F of Figure 1 the authors provide the quantification of the RFP negative clones area per disc pouch. Since the clones are generated via heat shock flippase, I think it will be more appropriate to compare the ratio of RFP negative clone area compared to RFP double positive area (twin spot). They have performed this kind of ratio quantification in Figure 2A-E.

> We would like to thank the reviewer for the suggestion. A graph quantifying the ratio of (*rer1*-/-/ twinspot) in wild-type, *rer1* mutant and rescue is now added in Figure 1J.

7. At what developmental stage the heat shock was done in Figure 2D, in order to have 120hrs clones (5 days). Do the larvae present a developmental delay? Could the authors provide details for the flyfood that was used for these experiments.

> The heat shock was given at 48 hrs after the egg laying. We have observed that in contrast to the control larvae, some larvae harbouring *rer1* mutant clones show a delay and can reach till 120 hrs (AHS). We have now clarified this in the result section (Page 5). Furthermore, we have provided the fly food recipe in the material and methods (page 12, *Drosophila* culturing).

8. In panel Figure 2E in the Y axis the symbol (%) is misleading. The twin spot is larger than the clone therefore the percentage of the ratio must be higher than 100%. For example in 120hrs the 20% ratio that is depicted, gives the impression that twin spot is smaller than the clones.

> Thank you for the suggestion. We have re-plotted the graph with the ratio of *rer1-/-* / twinspot instead of twinspot/*rer1-/-* to show the elimination of *rer1* mutant clones with the times (72, 96, and 120 hrs.), and also the "%" value in Y-axis of the graph is removed in the updated quantification (Figure 1E).

9. For the figure 2F-I, the authors have created rer1-/- clones in RpS3+/- background. They do not present the control experiments to make rer1+/+ clones (wild type) in RpS3+/- background, to investigate if rer1-/- clones perform worsen than rer1+/+ clones when they are next to RpS3+/- cells. I do not find informative the panel 2H. It would be meaningful, if we could compare this with the control situation, where they have wild type cells next to RpS3+/-. In that case they could compare if Dcp1 in RpS3+/- cells is different when the cells next to them have wild type rer1 (rer1+/+) or rer1-/- mutant. This experiment also shows that rer1-/- cells can still outcompete RpS3+/- cells.

> Thank you for the suggestion. We have now compared the survival of *RpS3+/-* cells in wild-type or *rer1* mutant background. As expected, we found that the wild-type cells are more efficient in outcompeting the *RpS3+/-* cells than the *rer1-/-* cells (Figure 2A-D). Furthermore, and more importantly, Dcp-1 was observed in the *RpS3+/-* cells abutting either wild-type or *rer1-/-* cells. These results suggest that, although less efficiently than wild-type, *rer1-/-* cells are able to outcompete the *RpS3+/-* cells. Moreover, possible reasons for the difference in the competitive fitness of these two cell types is now included in the discussion.

10. Have the authors tested if the heterozygosity of the null rer1 allele has any impact in Minutes. In the above experiments, the Minutes have also lost one copy of rer1 gene.

> As mentioned above, the *RpS3+/-* cells performed worse in wildtype background than in *rer1* mutant background, most likely due to lower fitness of *rer1-/-* cells as compared to the wildtype cells. However, whether the heterozygosity of *rer1* mutation also contributed to the elimination of *RpS3+/-* cells, could not be determined in these experiments.

11. In legend 2F they mention"hs-FLP-induced heterozygous RpS3+/– clones (GFP-positive cells) juxtaposed to rer1–/– cells (GFP-negative), and immuno-stained for the anti-cleaved Dcp-1.". According to their genotype, using the flippase they create rer1-/- clones in RpS3+/- background (the twin spot of these clones were RpS3-/-, rer1+/+, but the cells do not survive since RpS3 is an essential protein). Therefore, the most accurate description is " hs-FLP-induced rer1–/–clones (GFP-negative) juxtaposed to heterozygous RpS3+/– cells (GFP-positive cells), and immuno-stained for the anti-cleaved Dcp-1." They use also the term "RpS3+/- clones" in the text. It should be RpS3+/- background

>Thank you for the comment. We have edited the legends to include *RpS3+/-* background, however, we are using '*RpS3+/- cells*' to explain the juxta-positioning.

12. Comment for Figure 4A: Could the authors provide another disc with rer1-/- clones, where they will also depict p-eIF2a levels in comparison with DAPI staining (since p-eIF2a levels are cytoplasmic)? For example, in Panel 4B'' we can see higher levels of p-eIF2a in the top-edge of

the Figure where there is no rer1-/- cells. Maybe with the 72 hours AHS mitotic clones, which are bigger in size, the increase in p-eIF2a in rer1-/- mutant clones is more prominent. Indeed, in Figure 5B the increased levels of p-eIF2a in rer1-/- clones is more prominent than in 4A.

> We have updated this figure (new Fig 3) and we now provide 96 hrs *rer1-/-* clones along with the rescue. We find that *rer1-/-* mutant clones show higher *p-eIF2α* as compared to the neighboring cells with similar nuclear position.

13. In Figure S5 could they correlate the DHE staining with the nuclei position. In panel B''' there is an area in the anterior compartment with increased DHE, which actually is mainly cytoplasmic, which could be responsible for this. I kind of agree that it seems that DHE is increased in posterior compartment, but I would like to compare different areas of the two compartments with the same nuclei positions.

> We have reanalyzed the data and have now provided enlarged images comparing DHE levels in cells at similar nuclei position (S6B and D Fig).
