# Supplementary material for: Maintenance of proteostasis by Drosophila Rer1 is essential for competitive cell survival and Myc-driven overgrowth
Source: PLoS Genet. 2024 Feb 26;20(2):e1011171. doi: 10.1371/journal.pgen.1011171 (PMC10919865; doi:10.1371/journal.pgen.1011171)
Supplement: Attachment — Submitted filename: Reviewers Responses PLOS-Genetics minor revision v2.pdf [file pgen.1011171.s015.pdf]

Response to Reviewers' Comments

PGENETICS-D-23-00557R1

Comments to the Authors:
Please note here if the review is uploaded as an attachment.

Reviewer #3: Dear Paul, Umarvaish et al,
I find the responses to the reviewer's questions detailed and satisfactory. Thank you.
I have some minor concerns regarding the role of Rer1 in Myc supercompetition.

>We thank the reviewer for the encouraging comments and suggestions, which have further improved the manuscript.

Minor concerns:
A) Could the authors explain why they were unable to perform the experiment to analyze Myc-driven overgrowth in flies with an extra copy of the rer1 gene (GFP-rer1)? Did the larvae with the appropriate genotype (AFG/hs-FLP; UAS-Myc/GFP-rer1; UAS-GFP/+) die? Were they unable to retrieve clones? I find important for the field to present (or at least mention) the result of Rer1 overexpression.

> We attempted to analyze the impact of Myc overexpression in GFP-rer1 flies, using the AFG - mediated clones. Thus we co-expressed Myc with RFP (AFG/hs-FLP; UAS-Myc/GFP-rer1; UAS-KDEL-RFP/+) aimed to identify Myc-overexpressing (RFP+) cells in the GFP-Rer1 background. However, these larvae showed severe developmental delays post clone induction, failing to reach the third-instar stage. Similar delays were observed with the co-overexpression of Myc and Rer1 in the AFG clones (AFG/hs-FLP; UAS-Myc/UAS-rer1; UAS-GFP/+).

We believe a comprehensive evaluation of the possible synergistic effect of Myc and Rer1 overexpression require further detailed investigation using different overexpression tools and by also including temporal control using temperature sensitive Gal80. However, these aspects are currently not within the revision time and the scope of the present work.

B) Please provide some details and clarifications of the experiment that is provided in the Figure 9 and gives better support on the role of Rer1 in Myc supercompetition.

More specifically:
1) Could the authors provide the details on the heat shock time and dissection time in the experiment Fig. 9A-B.

>Apologies for this oversight. We inadvertently missed adding this information; The larval heat shock was administered at 48 hrs AEL for 60 mins, followed by dissection 72 hrs after heat shock. This information is now included in the materials and methods and figure legends.

2) Is the p-eIF2a ratio in Fig 9C increased in AFG::Myc, GFP, rer1+/- experiment compared to AFG::Myc, GFP due to the decrease of p-eIF2a in rer1+/- background cells? Is the ratio increased because the the Myc, rer1+/- cells have increased p-eIF2a? The images provided in the pdf version are not clear and they give the impression that the p-eIF2a staining is similar in AFG::Myc cells and in AFG::Myc, rer1+/- cells.

>We have not detected any difference in the p-eIF2a levels in rer1+/- and WT (rer1+/+ cells). For example, please check figure 3A; compare p-eIF2a in the twin spot (rer1+/+) and background rer1+/- cells. Moreover, as the loss of Rer1 leads to higher p-eIF2a, it is highly unlikely that rer1+/- cells in AFG::Myc, GFP, rer1+/- genotype will have lower p-eIF2a than normal cells. Thus, our data suggests that the p-eIF2a levels in AFG::Myc, GFP, rer1+/- are higher due to increased stress in Myc-overexpressing rer1+/- cells as compared to wild type.

We apologies for the image quality. We have now updated figure 9 with better quality images (please also see below).

3)Have the authors performed control clones in WT and in rer1+/- background, meaning AFG without over-expressing Myc. Do the clones look smaller in rer1+/- background?

> We have analyzed GFP expressing AFG control clones in both WT and in rer1+/-. We observed that growth of GFP clones was similar in both conditions and no effect on p-eIF2a levels was observed due to GFP expression. This data is now presented in the new supplemental figure S11 and mentioned in the results section (last paragraph).

4) If the authors have discs that the Myc, GFP cells have not undertaken the whole disc would be useful. Also show discs with similar size (the 9A disc looks bigger than 9B).

> We have performed this experiment only at 72 hours post clone induction by which time a large part of the discs is covered by the Myc-overexpressing clone. However, we have updated the images (as also shown below) which now show disc with slightly larger GFP-negative for better analysis of p-eIF2α levels. We would also like to point out that we have noticed slightly smaller discs when expressing Myc, GFP in rer1+/-, possibly due to death of cells visibly showing strongly upregulated p-eIF2α.

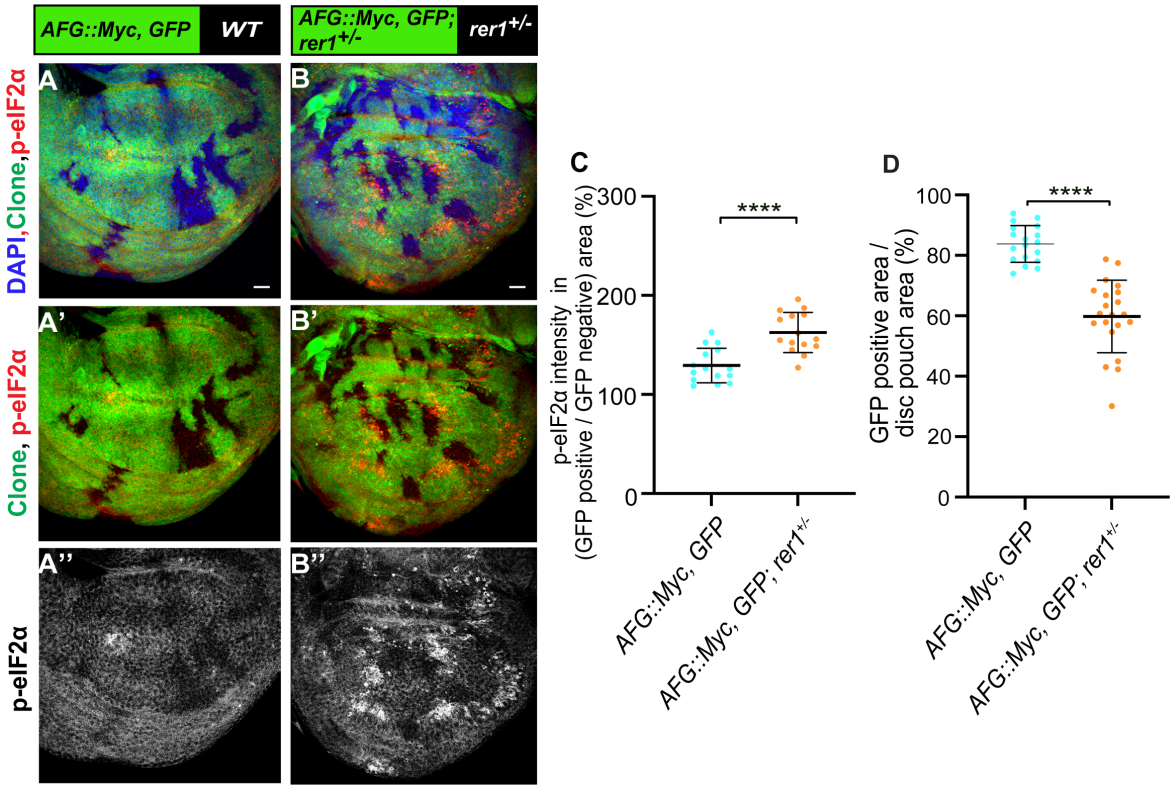

C) Please mention that also in Ochi et al, PERK depletion suppressed the elimination of wol clones (wol is involved in the glycosylation of proteins in the ER), of Hel25E clones.

>Thank you for this suggestion. We have added the following sentence and also included RpL14 (please also see discussion, fourth paragraph). “In contrast, blocking p-eIF2α by PERK depletion was shown to suppress the competitive elimination of *wollknaeuel* (*wol*) mutant clones (wol is involved in the glycosylation of proteins in the ER), *Hel25E* mutant clones and *RpL14+/-* cells [15]” i.e.(Ochi et al., 2021).
